# Effects of Cocaine on Maternal Behavior and Neurochemistry

**DOI:** 10.2174/157015912799362760

**Published:** 2012-03

**Authors:** Benjamin C Nephew, Marcelo Febo

**Affiliations:** 1 Department of Biomedical Sciences, Tufts University Cummings School of Veterinary Medicine, Grafton, MA, USA; 2 Department of Psychology, Northeastern University, Boston, MA, USA

**Keywords:** Cocaine, maternal behavior, aggression, vasopressin, oxytocin, dopamine, serotonin, depression, anxiety, stress.

## Abstract

Drug addiction is a chronic relapsing disorder that involves drug seeking and abuse despite the negative social and health consequences. While the potential effects of cocaine on child development have been extensively studied over the last 30 years, few researchers have focused on the effects of cocaine on maternal behavior, which includes offspring care and maternal aggression towards an unfamiliar individual. In humans, maternal cocaine use can lead to child neglect, abuse, and disrupt the mother-child bond. While it has been argued the developmental effects of maternal cocaine use on children were initially overstated, it is clear that disruptions of typical maternal behavior (i.e. postpartum depression, anxiety disorders) are detrimental to the physical and emotional health of offspring. Cocaine use in mothers is commonly associated with psychological disorders, including depression and anxiety, and it is postulated that many of the negative effects of maternal cocaine use on offspring are mediated through changes in maternal behavior. This review will summarize research on cocaine and maternal behavior in animal and human studies, discuss potential mechanisms, and suggest therapeutic strategies for treating cocaine-affected maternal behavior which may improve the physical and behavioral health of both mother and child. The primary objective is to stimulate future communication, cooperation, and collaboration between researchers who use animals and humans to study cocaine and maternal behavior.

## INTRODUCTION

Drug addiction is a chronic relapsing disorder that involves drug seeking and abuse despite the negative social and health consequences. There is a body of literature indicating that maternal-offspring interactions are affected by cocaine dependence in the mother, which is highlighted in the recent review of maternal cocaine addiction by Strathearn and Mayes [1]. This is especially interesting in light of the fact that maternal-infant attachment is so strong and the rewarding properties of offspring are dominant in the mother. However, mothers recovering from cocaine addiction may experience difficulties in their maternal roles as effective caregivers. Some studies cite reduced mother-child play interactions, low-self esteem, emotional neglect, lack of maternal identity, increased hostility towards their own child, and inability to cope with stress [2-5]. Although socioeconomic factors are involved, neurobiological adaptations resulting from chronic cocaine abuse also may have lasting effects on the maternal brain. While the potential effects of cocaine on child development have been extensively studied over the last 30 years, few researchers have focused on the effects of cocaine on maternal behavior, which includes offspring care and maternal aggression towards an unfamiliar individual. In humans, maternal cocaine use can lead to child neglect [6], abuse [7], and disrupt the mother-child bond [8]. While it has been argued the developmental effects of maternal cocaine use on children were initially overstated [9], it is clear that disruptions of typical maternal behavior (i.e. postpartum depression, anxiety disorders) are detrimental to the physical and emotional health of offspring (for review, see [10]). Cocaine use in mothers is commonly associated with psychological disorders, including depression and anxiety, and it is postulated that many of the negative effects of maternal cocaine use on offspring may be mediated through changes in maternal behavior.

In addition to the few clinical studies of maternal cocaine abuse, the use of a maternal rodent model has provided valuable insight on this topic. Studies of the effects of cocaine on rodent maternal behavior have included investigations of both maternal care and maternal aggression. Maternal rats provide warmth, grooming, and milk for offspring survival and growth, while simultaneously displaying maternal aggression that serves to protect offspring from conspecifics [11, 12]. Rats are an ideal model for investigating multi-generational genetic and epigenetic effects of cocaine, as well as non biological forms of maternal behavior, such as foster care and day care. Cocaine exposure that disrupts maternal care or aggression may have negative effects on off-spring physical or behavioral health. This review will summarize research on cocaine and maternal behavior in animal and human studies, discuss potential mechanisms, and suggest therapeutic strategies for treating cocaine-affected maternal behavior which may improve the physical and behavioral health of both mother and child.

## METHODS

A search for “cocaine maternal behavior” on Pubmed generated 300 citations, and searching for “cocaine maternal aggression” generated 25 citations. For this review, we selected studies from these lists which were focused primarily on cocaine and maternal behavior and/or, and not on the effects of other drugs of abuse or the developmental effects of cocaine on offspring. Potential mechanisms for the effects of cocaine on rodent maternal care and aggression were identified in the selected studies on cocaine and maternal behavior. The list of therapeutic targets was generated by a review of the maternal behavior and cocaine addiction literature. The 3 targets with the most overlap in maternal behavior and cocaine addiction were serotonin (5-HT), oxytocin (OXT), and vasopressin (AVP). Studies were subdivided into the 6 following sections: Cocaine and Rodent Maternal Care, Mechanisms for the Effects of Cocaine on Rodent Maternal Care, Cocaine and Rodent Maternal Aggression, Mechanisms for the Effects of Cocaine on Rodent Maternal Aggression, Cocaine and Human Maternal Behavior, and Potential Therapeutic Targets.

## COCAINE AND RODENT MATERNAL CARE

Over the past 30 years, researchers have investigated the effects of prior adult exposure to cocaine, as well as gestational and lactational treatments. Cocaine treatment of adult female rats prior to mating increases subsequent maternal care [13-16]. Females treated with cocaine as adults are faster to retrieve pups, and spend more time grooming them. It has been postulated that this effect is due to a priming of the central reward pathway. However, additional studies are needed to determine if prior adult exposure affects offspring health. Early studies of gestational cocaine using relatively low doses (<30mg/kg) concluded that cocaine had minimal effects on the onset of maternal care, and no effects on established maternal care [17]. Studies using higher gestational doses have reported significant disruptions in nest construction [18, 19], retrieval [20], and ongoing maternal behavior [19, 21-23]. Kinsley *et al*. (1994) noted that pups would be retrieved, but then the dams would ignore them, suggesting that cocaine interferes with the organization of effective maternal behavior. The majority of studies showing significant behavioral effects of gestational cocaine involve doses > 30mg/kg per day. Interestingly, behaviorally effective gestational doses are higher than behaviorally effective doses used in the adult exposure studies (15mg/kg). This might suggest that the gestational effects of cocaine may arise from teratologic actions that could affect pup health and/or behavior in addition to impacting maternal behavior. There is evidence that maternal rats treat cocaine exposed pups differently, and the overall effects of cocaine on maternal behavior may be the result of a dam-pup interaction [21]. To test whether withdrawal from cocaine mediates the effects of gestational treatment, dams were treated with cocaine during gestation and lactation and compared to dams that were only treated during gestation. Both groups expressed impaired levels of maternal behavior, indicating that the deficits were not dependent on changes during withdrawal [22]. Gestational cocaine not only disrupts maternal behavior in the treated dam, it also disrupts the maternal behavior expressed by the adult offspring [21]. These intergenerational effects were attributable to both gestational cocaine exposure and rearing experience.

Exposure to cocaine during lactation also impairs maternal care. Zimmerberg *et al*. reported that chronic cocaine during lactation decreases pup retrieval, and sniffing and licking of pups sixty minutes following injection [24]. Acute cocaine on postpartum day 5 or 6 disrupts crouching and increases latencies to contact pups, retrieve, lick, group, and crouch over them [23]. As with gestational treatments, these two studies suggest that cocaine’s major effects seem to disrupt ongoing maternal behavior. Cocaine treated females have an attenuated interest in pup care, and are not highly motivated to express maternal care. These effects of acute cocaine on maternal care are present as early as day 1 of lactation [19]. The disruption of maternal care at this point is especially dangerous to offspring health, as this is when altricial rodents are most dependent on their mother. Targeted injection of cocaine into the medial preoptic area (mPOA) or nucleus accumbens (NA) significantly disrupts maternal care, indicating that cocaine does not need to act on multiple neural cites to modulate maternal care [25].

## MECHANISMS FOR THE EFFECTS OF COCAINE ON RODENT MATERNAL CARE 

A few studies relying on targeted injection or neural activity markers have identified several key nuclei where cocaine may be acting to modulate maternal behavior (for review, see [26]). There are specific neural circuits that are involved in the display of parental patterns across many species. One such set of structures are involved in reward/pleasure seeking behavior and has been coined the reward system. It is comprised of neurons in the ventral tegmental area (VTA) that synthesize the neurotransmitter dopamine and that project to the nucleus accumbens and prefrontal cortex. In turn, the VTA receives direct and indirect projections from both of the latter regions in addition to excitatory glutamatergic inputs from hippocampal and amygdalar nuclei. The VTA also reciprocally communicates with the mPOA of the hypothalamus. Connections between the VTA-mPOA have been shown to be involved in the display of maternal behavior [27]. Circuits from higher cognitive centers such the medial prefrontal cortex (mPFC) presumably modulate the appetitive aspects of these naturally rewarding behaviors in rats. A study of c-fos activity following exposure to cocaine and pup cues in a conditioned place preference design revealed significant increases to both cues in the medial prefrontal cortex (mPFC) and basolateral amygdala. C-fos activity was increased in the mPFC, nucleus accumbens, and basal amygdala with cocaine cues compared to pup cues or control treatment [28]. These nuclei are involved in motivational processing. When dams conditioned to both cocaine and pups are given a choice in a conditioned place preference study, they chose the pup cue side on day 8 of lactation, but chose the cocaine cue side on days 10 and 16 [29, 30]. This suggests that the motivation to express maternal care is stronger than the motivation for cocaine during early lactation and presents a useful paradigm for investigating the mechanisms controlling maternal care across lactation. An early functional MRI study [31], revealed that suckling stimulation from pups can enhance neuronal activity in the reward system of the mother, but cocaine has the opposite effect of shutting down these regions, perhaps due to changes in the mesocorticolimbic dopamine system. To summarize, acute administration of cocaine and chronic treatments during gestation and lactation are known to negatively affect maternal care in rats, and this is due to the presence of cocaine in the bloodstream and its direct actions on brain chemistry. However, relapse to drug seeking and taking behavior arises during periods of abstinence and is believed to arise from long-term neuroadaptations within specific limbic and cortical circuitries. 

To explore long-term neural correlates of cocaine sensitization and withdrawal, control dams and dams sensitized to cocaine prior to mating were imaged for their BOLD responses to suckling stimulation from pups [13]. Consistent with previous work [31], it was observed that suckling activated areas of the reward system (VTA-Accumbens-mPFC). However, cocaine sensitized dams showed a severely blunted BOLD signal response in the prefrontal cortex, particularly the orbital-infralimbic regions. It appears that reduced prefrontal cortical BOLD signal responses may be a long-term consequence of cocaine sensitization earlier in life. These findings are consistent with the human literature showing reduced metabolism in the prefrontal cortex of cocaine dependent subjects [32]. It was also observed that extracellular levels of dopamine in the medial PFC, as measured by *in vivo* microdialysis, are elevated by exposure to pups, but no differences were observed between control and cocaine sensitized mothers [13]. Therefore, dopamine release mechanisms do not seem to be affected. However, several previous studies have indicated that dopamine is involved in maternal behavior.

Inhibiting central dopamine activity around parturition impairs the expression and retention of typical maternal care [33], and long-term reuptake inhibition of dopamine during gestation can increase nesting and pup grooming [34]. Dams with dopamine depleting lesions in the ventral striatum have longer retrieval latencies [35, 36], and dopamine receptor blockade in the nucleus accumbens inhibits retrieval and licking, but may increase nursing behavior [37]. However, the deficits in maternal care following ventral striatum lesions are eliminated by a separation-induced augmentation of maternal motivation [35], indicating that other mechanisms can compensate for the behavioral effects of these dopamine depleting lesions. Dopamine depleting lesions of the VTA during pregnancy completely block maternal care, but treatment 8 weeks prior to parturition has no effect [38]. These results suggest that dopamine plays an active role in the development of maternal care during gestation, but other mechanisms may compensate for previously impaired dopamine activity in the VTA. Specific injections of dopamine antagonists impair maternal behavior, although this effect may be mediated by depressed locomotor activity [39], as dopamine antagonists disrupt the locomotor associated components of maternal behavior [36, 40, 41]. Given all the studies supporting the hypothesis that dopamine is positively associated with maternal care, the reported inhibitory effects of gestational cocaine on maternal behavior [22] are somewhat unexpected, as active responding to pups is correlated with DA release in the ventral striatum [42]. It is possible that dopamine is depleted by gestational cocaine in other behaviorally relevant nuclei, or that related changes in central oxytocin OXT or AVP activity are responsible for the altered maternal behavior.

OXT is a likely candidate for the effects of cocaine on maternal behavior due to its importance in the control of social behaviors in many species [43-46]. The involvement of oxytocin in maternal care specifically parallels similar reports of the importance of OXT in the formation of other types of social bonds. Cocaine treatment prior to mating, which results in elevated maternal care [14], may prime the brain for elevated OXT release in response to pup stimuli during lactation. However, it is unclear how OXT is specifically related to cocaine induced changes in maternal behavior, as increased OXT has been recorded following chronic cocaine treatments that both decrease maternal care [47], as well as increase maternal care [15]. Pre-mating cocaine exposure increases maternal care when administered in the maternal care testing environment and is associated with a trend for increased OXT in the hypothalamus [15]. One possible explanation for the difference in these studies is the timing of the cocaine treatment, as McMurray *et al*. administered cocaine during gestation, and Petruzzi *et al*. gave cocaine prior to mating. It may be that cocaine modulated other hormones in addition to oxytocin when administered during gestation, as oxytocin is generally thought to have a stimulatory effect on maternal care. In support of this hypothesis, gestational cocaine suppresses both maternal care and OXT levels in the ventral tegmental area, hippocampus, and MPOA during the first 2 days of lactation [48], as well as decreasing oxytocin receptors in the MPOA on day 6 [49]. Acute injection of cocaine during lactation depresses OXT in the MPOA on postpartum day 1, and increases OXT on postpartum day 6 in the amygdala [50]. The same treatments have been previously associated with decreases in maternal care and aggression on these days [51]. These results suggest that acute oxytocin during lactation has a stimulatory effects on maternal care, and an inhibitory effect on maternal aggression. In support of this hypothesis, McMurray *et al*. reported that the next generation dams raising their own litters displayed elevated aggression which was associated with suppressed OXT levels in the amygdala [52]. While it is clear that OXT can modulate maternal care, further study is needed to determine the mechanism controlling these effects. Another valuable target of studies on the effects of cocaine on maternal behavior is AVP. 

Although vasopressin is typically associated with its physiological effects on water balance, blood pressure, and the stress response, earlier studies on OXT and recent reports on the behavioral effects of vasopressin suggests that it is an important component mediating the display of maternal care. Changes in central vasopressinergic activity affects the induction [53] display [54-56] and retention [57] of maternal care. There is also evidence that vasopressin is involved in sensitization to cocaine [58-60] and withdrawal [61, 62], and based on both the maternal behavior and the addiction literature, it is hypothesized that cocaine exposure affects maternal behavior through disruptions of central vasopressin signaling.

## COCAINE AND RODENT MATERNAL AGGRESSION

Treatments that affect maternal care often affect aggressive responses as well, and cocaine exposure follows this pattern. Chronic pre-mating adult exposure heightens the aggressive responses to a male intruder [14, 15]. These effects may be dependent on an interaction between cocaine and a conditioned response to the environment where cocaine was administered [15], and support the hypothesis that prior cocaine use may have long-term effects on maternal behavior. Cocaine treatment on gestation days (GD) 8-20 increase maternal aggression compared to saline controls. These effects were specific to aggression, as neither nest building nor pup retrieval were affected by the cocaine treatment [63]. However, several other studies using similar treatments have documented deficits in both maternal care and aggression [19, 22, 51]. There is evidence that elevated levels of aggression can be transmitted to future offspring through maternal behavior, as cocaine-free pups raised by aggressive dams treated with cocaine as adults are also more aggressive [64]. This suggests that the effects on offspring are behaviorally mediated and supports the conclusions of human studies which underscore the significance of the maternal behavior mediated effects of cocaine on child behavior. Further investigation of elevated maternal aggression following gestational cocaine concluded that the intensification was not due to withdrawal effects, as there was increased maternal aggression following both gestation only treatment and gestational and lactational treatment [22]. The aggressive effects of gestational cocaine seem to require doses of at least 30 mg/kg, as lower doses had no effect on aggression [65]. It is possible that these higher doses trigger a robust behavioral sensitization that is necessary for the aggression effects to be consolidated. In addition to aggression towards a novel intruder, gestational cocaine treated dams are also more defensive towards an inanimate object [66], suggesting that cocaine may heighten the nonspecific behavioral response to threatening stimuli. However, a recent report concludes that chronic gestational cocaine has no effect on maternal aggression, but intermittent cocaine decreases maternal aggression [52]. Further study is needed to explore the behavioral and neurochemical differences between chronic and intermittent treatments. In contrast to most of the studies of pre-mating and gestational cocaine treatment, acute cocaine administered during lactation decreases maternal aggression [19, 51]. Based on the studies to date, it appears that cocaine exposure can modulate maternal aggression, but the direction of the effects may be dependent on the dose, period and/or schedule of exposure to cocaine. 

## MECHANISMS FOR THE EFFECTS OF COCAINE ON RODENT MATERNAL AGGRESSION

One potential mechanism for the effects of cocaine on maternal aggression is through the modulation of central oxytocin (OXT). Cocaine induced changes in central OXT may mediate the increase in aggression, as maternal aggression has been associated with decreased oxytocin activity in the amygdala [47, 52] and mPOA [67]. Gestational cocaine treatment elevates maternal aggression on day 6 and results in decreased OXT, increased OXT receptor binding density, and decreased OXT receptor affinity in the amygdala, [68]. This study also reported decreased OTR binding in the VMH and CeA, and the VMH has previously been associated with non-maternal aggression [69-71]. Taken together, these reports suggest that central OXT activity inhibits maternal aggression. However, some studies have reported no association between cocaine induced maternal aggression and OXT [65], and it is possible that the recorded changes in OXT following cocaine treatment are more related to simultaneous alterations in maternal care. Since maternal care and aggression are temporally coexpressed, careful manipulative studies are needed to determine the importance of OXT in the expression of each behavior. In contrast to the chronic gestational treatment, the suppression of aggression by acute cocaine during lactation has been associated with an increase in amygdalar OXT [50]. Reduced central OXT activity has been previously associated with increased maternal aggression in several studies of non-cocaine treated rats [50, 66, 72-74], but other studies conclude that OXT stimulates maternal aggression [75-78]. In their 2002 review of maternal aggression, Lonstein and Gammie conclude that the role of OXT in maternal aggression is not clear [140]. Based on the results of the most recent studies, this conclusion is still accurate. Other potential candidate neurohormones for the control of maternal aggression are dopamine, 5-HT, and AVP.

Gestational cocaine may alter maternal aggression through cocaine’s effects on dopamine activity. With respect to non-cocaine associated maternal aggression, it appears that dopamine plays an inhibitory role, as the destruction of cells in the ventral tegmental area increase maternal attacks [36]. However, dams given 30mg/kg cocaine during gestation are more defensive towards an inanimate object and have elevated dopamine levels relative to saline controls [79]. In contrast, dopamine uptake inhibition depresses maternal aggression on day 6 of lactation [34]. This inhibitory role for dopamine is indirectly supported by the attenuation of aggression following acute cocaine [19] and dopamine uptake inhibition [34] during lactation. It is postulated that the direction of dopamine’s effects on maternal aggression may be dependent on the specific nuclei and timing of the dose. Studies that comprehensively assess DA activity in multiple nuclei may provide useful insight into DA’s role in maternal aggression.

5-HT 2A/2C receptors in the dorsal PAG are involved in the inhibition of female aggressive behavior, and this effect is not mediated by locomotor effects [80]. It has been postulated that the excitatory effects of the blockade of 5-HT reuptake on maternal aggression may be mediated by changes in central OXT [34]. The effects of this increase in 5-HT on maternal aggression may be mediated by a decrease in central OXT activity, as this treatment also decreases OXT binding affinity in the amygdala. While 5-HT reliably decreases aggression in male models, this finding of opposite effects in females parallels the related hypothesis that the control of maternal aggression by AVP is divergent from the established mechanism for male aggression. In several rodent species, AVP increases aggression and 5-HT decreases aggression in males [81-85]. There is now evidence that endogenous AVP depresses maternal aggression through actions at the V1a receptor [56, 86] and multiparous rats with lower AVP expression in the paraventricular nucleus are more aggressive compared to primiparous females with their first litter [74]. Interestingly, in humans, central 5-HT activity is inversely correlated with aggression in males, but not in females [87]. Although AVP and 5-HT may mediate both male and maternal aggression, the direction of the effects may be sex dependent. Simultaneous study on 5-HT, OXT, and AVP is needed to determine the relative roles of each of these neurohormones.

## COCAINE AND HUMAN MATERNAL BEHAVIOR

One important issue to consider when assessing the literature on the effects of cocaine on maternal behavior and associated child behavioral issues is the method of reporting. Most studies relying on parent reporting of behavioral issues do not find significant effects of prenatal cocaine exposure. In comparison, the majority of studies relying on teacher reports conclude that cocaine exposure does disrupt offspring behavior. Some mothers are reluctant to complain about a child’s behavior due to fear of losing custody. This confound must be carefully considered when assessing the literature, and independent reporting methods are more reliable.

In a thorough review of the effects of cocaine on offspring development and behavior, Frank *et al*. noted that prenatal cocaine exposure may result in impaired attentiveness and emotional expressivity in offspring [9]. Several researchers have noted that cocaine only has significant effects on offspring behavior if maternal behavior is disrupted. A comparison of parental and non-parental care of cocaine exposed toddlers supports this conclusion, as cocaine exposed toddlers in non-parental care had improved development compared to those still in parental care [88]. This study suggests that improvements in care giving may improve the outcome of children born to cocaine using mothers. Some of these potential improvements include direct intervention with kids, supporting women in recovery, and helping women to improve parenting skills. Numerous studies over the last 10 years have independently concluded that the negative effects of cocaine on offspring are mediated by psychiatric disorders which lead to impairments in maternal behavior. This maternal behavior mediated mechanism may potentially affect millions of offspring, as psychiatric disorders which impair parental behavior are relatively common in substance using populations, with antisocial disorders being most prevalent [89]. However, substance abuse can also impair maternal behavior independent of comorbid psychological issues [90].

In one long-term study, cocaine use during pregnancy impaired mother-child interactions at 3 years, especially when cocaine use was continued at this time. These impairments included increased maternal hostility, decreased maternal confidence, and poor quality parental instruction [4]. Cocaine using mothers are less attentive and interactive with their infants during the first 6 months [91, 92]. Although active users may have the most significant deficits in maternal behavior, recovering cocaine addicts also exhibit impaired maternal care [2, 3]. Mothers addicted to cocaine show more negative engagement with their babies [93, 94], and recent studies suggest that the exposure to various environmental stressors can modulate the negative effects on child behavior. Substance abusing mothers suffer from elevated stress, and are more likely to abuse and neglect their children [95]. Furthermore, addicts find parenting more stressful, and are more likely to use ineffective parenting techniques [96]. Maternal drug use in a stressful environment may have a particularly negative impact on parenting. As the number of environmental risk factors (depression, domestic abuse, psychiatric symptoms, absence of significant other) increases, a substance abusing mother may be overwhelmed and have little time for effective parenting [97]. While the increase in environmental risk factors in cocaine using mothers is associated with child abuse and neglect [98], additional environmental stress is not required for cocaine use to significantly impair maternal behavior [99-101]. It has been suggested that the perception of parenting stress has a more negative impact on neonatal behavior and infant temperament than drug exposure history [102]. Maternal psychological issues may exacerbate the effects of cocaine on the mental and motor development of the child, as parental distress severity is an independent predictor of child mental health [103]. Taken together, these studies support the hypothesis that cocaine use impairs maternal behavior, and many of the common risk factors associated with drug abuse intensify this impairment. One of the most significant risk factors is maternal depression.

Several reports suggest that there is a link between cocaine use, psychological health, and impaired maternal behavior [89, 104-106]. The most common psychiatric illness associated with impaired maternal care in substance abusing mothers is depression. One study that reported no effect of cocaine on child behavior did find that depression in both the cocaine exposed and control groups was associated with behavioral problems [107]. The authors recommend that mothers should be carefully screened for depression, independent of substance abuse history. Maternal drug use predicts unresponsive and negative maternal behavior, but these effects are mostly due to comorbid maternal psychopathology [89]. Another study concluded that prenatal cocaine exposure is not predictive of child behavior at age 5, but maternal psychological health is strongly linked to child behavior [104]. Many of the negative effects of cocaine on maternal behavior may be mediated by depression.

In a study of attentiveness, cocaine-abusing mothers spent less time being attentive towards their children, and had more shifts in attention compared to non-cocaine using controls. However, these differences were found only at 3 months of age, and not at 6, 12, or 18 months. In fact, maternal attentiveness was more strongly related to current depression symptoms than addiction severity in the cocaine abusing mothers, once again suggesting that the negative effects of cocaine are mediated through its depressive effects [105]. Both postnatal cocaine use and maternal depression are reliable predictors of maternal insensitivity during mother-child interactions at 4-8 weeks of age [106]. Some researchers argue that prenatal cocaine exposure has no impact on the strong association between parenting stress at 4 months and child externalizing behavior at 36 months [108], suggesting that postnatal use is more disruptive to maternal behavior. However, it should be mentioned that some studies finding no direct effect of cocaine on child behavior may be confounded by the assessment of children that are still with their parents, with the exclusion of more severely exposed children that were removed from the home. When parental behavior is assessed in detail, cocaine abusing mothers suffer from lower parental scores, poor parental attitude, and poor mother-child interaction [109]. There is also evidence that infant directed sensitivity is lower in cocaine using mothers at 6.5 and 12 months, and maternal sensitivity to their infant is inversely correlated to the concentration of cocaine metabolites in the mother [110]. In contrast, one study actually reported that prenatal cocaine exposure may suppress the substantial negative effects of postpartum depression on infant neurobehavior, as the infants of cocaine using mothers were less stressed and excitable [111]. These data contrast with statistics that clearly indicate that cocaine using mothers are more likely to lose custody of their children.

High levels of cocaine use are strongly associated with failure to maintain custody of children due to neglect and/or abuse [6]. In a study of 115 cocaine using mothers and 105 non-using mothers, 19% of cocaine mothers lost custody within 1 month of birth, compared to 0.02% of non-using mothers [112]. While there are obvious risks associated with keeping children with their addicted mothers, removing children to foster care is most likely not beneficial to the mother’s recovery from addiction. Crack using mothers without kids are more likely to use than mothers with custody of their children [113], supporting the hypothesis that children have a high incentive value [114]. A following study found that family-focused interventions that address family conflict and communicate disapproval of substance abuse may lower intergenerational risk transmission for addictive behavior. In support of addicts maintaining custody, cocaine using mothers develop normal maternal-fetal attachment during gestation [115], maternal substance use does not seem to affect the perception of the infant [102], and children become markers for the success of recovery for drug-addicted mothers [113]. It is important to minimize stress and environmental risk factors affecting a mother struggling with cocaine dependency, as it is clear that parenting stress and an elevated number of risk factors play a large role in mediating the negative effects of cocaine on maternal behavior. It is possible that removing children from cocaine abusing mothers may lead to increased cocaine exposure and parenting of subsequent offspring due to an increase in stress-mediated relapse of the mother.

## POTENTIAL THERAPEUTIC TARGETS

The human and rodent literature suggests that the focus for any treatment method should be the cessation of cocaine use and the psychological health of the mother. Given the high rate of comorbidity between addiction and psychiatric disorders, this would be advisable for both virgin and maternal addicts. In virgins, this type of treatment would be a preventative measure for the maladaptive effects of cocaine use and associated psychological issues on subsequent offspring. The few intervention programs that have included drug using mothers suggest that programs that treat both the child and the caregiver can improve child outcomes in families with cocaine exposed parents. The most effective treatments may be center or home-based, rather than based on the primary care provider [116, 117]. It has also been suggested that treating both mental health issues (addiction and comorbid psychiatric disorders) and parental deficits is critical to effective treatment [89]. Addicts who are treated for psychiatric disorders are less likely to employ ineffective parenting practices [90]. Unfortunately, most parents with psychiatric disorders do not receive appropriate treatment [90, 118].

Animal studies provide speculative evidence of potential mechanisms for the effects of cocaine on maternal behavior, but the frequency of conflicting studies suggests that further research is needed. Multiple studies in rodents have independently concluded that the roles of some neuropeptides, such as AVP and 5-HT, may have opposite effects in males and females. This is an important issue to consider when developing novel treatments for impaired maternal behavior in cocaine users, as many clinical studies focus on males, and rarely involve maternal subjects. Three of the most promising targets for the development of effective pharmacological treatments are OXT, AVP, and 5-HT.

While the exact role of OXT in cocaine disrupted maternal behavior is not clear, there is ample evidence that OXT is involved in the mechanism. Oxytocin’s involvement in the establishment of social bonds has been repeatedly demonstrated in both rodent and human models [43, 44, 119, 120]. OXT pretreatment facilitates the development of cocaine induced behavioral sensitization in rodents, and it is postulated that gestational and lactational cocaine use may impair maternal behavior in humans through OXT suppression. Cocaine using mothers have depressed plasma OXT levels, increased hostility and depression, and tend to hold their infants less frequently [94]. The suppression of plasma OXT may be linked to the disruption of mother-child bond and increases in child neglect and abuse. In abstaining mothers, OXT treatment during lactation may ameliorate the negative effects of cocaine use on maternal behavior and enhance attachment and beneficial maternal care. An interesting retrospective study would be to look at the parenting behavior of breast-feeding and non-breast feeding recovering addicts to investigate whether breast-feeding associated OXT release may improve cocaine disrupted maternal behavior.

Although AVP has not been thoroughly explored as a possible treatment target for addiction or psychiatric disorders, recent evidence suggests that this neuropeptide may be a promising target for stress-mediated depression and anxiety disorders [121-128], as well as have a role in the aversive effects of early withdrawal [61, 129, 130]. Rodent studies have indicated that it is involved in both the expression and retention of maternal behavior [55-57, 74, 86, 131]. Based on these roles, it is postulated that this neuropeptide may be a relevant target for treating cocaine induced disruptions in maternal behavior. Drug use and withdrawal are potent stressors [132-134], and acute stress elevates the level of AVP in the rat amygdala and hypothalamus [135, 136]. Furthermore, the activation of V1b receptors in male rats mediates anxiety and depressive behaviors [128, 137], and it has been suggested that the AVP V1b receptor is a relevant target for the prevention of heroine relapse [129]. Although AVP activity in the medial amygdala is positively associated with the expression [55, 56] and retention of rodent maternal care [57], it has inhibitory effects on maternal aggression through actions at the V1a receptor [56, 86]. Further study is needed to determine if withdrawal-mediated changes in central AVP affect maternal behavior and offspring health. Acute cocaine withdrawal results in elevated AVP mRNA levels in the rodent amygdala, and this withdrawal effect is blocked by naloxone, suggesting that the effect of cocaine on AVP mRNA is opioid receptor mediated [61]. Although these results are interesting, it should be noted that most studies use male rats, and the effects of cocaine withdrawal in lactating female rats could be different. However, AVP is behaviorally active in maternal rodents, and the modulation of central AVP activity during the withdrawal period may decrease perceived stress, directly improve maternal behavior, and/or decrease the likelihood of a relapse in humans. 

Central 5-HT activity is linked to changes in AVP, and may also be useful tool for treating deficient maternal behavior due its well-established role in depression. Several studies have noted that negative effects of cocaine exposure on maternal behavior are often mediated by symptoms of depression, and selective serotonin reuptake inhibitor treatment may enhance maternal behavior in depressed addicts. To confirm that depression symptoms are independent of addiction, treatment should begin after a period of abstinence in cocaine using mothers. It is hypothesized that treating both addiction and comorbid depression and/or anxiety disorders will result in a greater improvement in maternal behavior. It is likely that past or current exposure to a substantial stressor mediates the link between addiction, depression, and maternal behavior.

Women entering substance abuse treatment show signs of early life trauma that hinders their ability to carry forth normal lives [138]. Ultimately, any strategy to pharmacologically ameliorate craving for addictive substances and minimize stress in order to enhance maternal care under impoverished conditions will benefit from psychological intervention for coping with stress and early life trauma [139]. Studies specifically designed to investigate potential treatments for special populations, especially cocaine addicted mothers, are lacking. Therefore, current treatment paradigms are based on studies which do not directly investigate the effects of cocaine on maternal behavior. The presence of significant gender differences in both addiction to cocaine and responsivity to treatment underscores the need for studies of cocaine addiction and its long-term impact on maternal neurobiology and behavior. 

## SUMMARY AND CONCLUSIONS

This review presents animal and human studies of cocaine and maternal behavior with the primary aim of stimulating future communication, cooperation, and collaboration among researchers who use animals and humans to study cocaine and maternal behavior. A secondary aim is to stimulate general research interest in female cocaine addiction, which is lacking when compared to research on male cocaine addiction. Improvements in the prevention and treatment of female cocaine addiction have the potential to directly improve the lives of not only the maternal female, but her offspring as well. Recent investigations suggest that a focus on cocaine, stress, psychological disorders, and maladaptive maternal behavior may yield useful insights on the development of effective treatments for cocaine affected maternal behavior. Although there is a clear need for further studies focusing directly on the effects of cocaine on maternal behavior, three promising targets for the development of novel treatments for cocaine-impaired maternal behavior are OXT, AVP, and 5-HT.
